# Biochemical modulation of growth, lipid quality and productivity in mixotrophic cultures of *Chlorella sorokiniana*

**DOI:** 10.1186/2193-1801-1-33

**Published:** 2012-10-06

**Authors:** Momocha Ngangkham, Sachitra Kumar Ratha, Radha Prasanna, Anil Kumar Saxena, Dolly Wattal Dhar, Chandragiri Sarika, Rachapudi Badari Narayana Prasad

**Affiliations:** 1 Division of Microbiology, Indian Agricultural Research Institute (IARI), New Delhi, 110012 India; 2 Centre for Lipid Research, CSIR-Indian Institute of Chemical Technology, Hyderabad, 500 007 India

**Keywords:** Carbon metabolism, Chlorella, FAME, Glucose, Lipids, Reducing agent

## Abstract

The genus *Chlorella* is a widely employed microalga for biodiesel, as it can be grown using photo/mixo/heterotrophic mode of cultivation. The present investigation was undertaken with the hypothesis that addition of different substrates (amino acids, carbon sources, vitamins) along with reducing agents may aid in diverting Acetyl CoA to malonyl CoA or fatty acid biosynthesis, under mixotrophic conditions in *Chlorella sorokiniana.* Preliminary investigations undertaken with two reducing agents individually (sodium thiosulphate and methyl viologen) along with selected substrates revealed the promise of sodium thiosulphate (1%) in enhancing lipid accumulation significantly. Further, the role of inclusion of twelve substrates and sodium thiosulphate revealed that supplementation with tryptophan (0.1%) recorded 57.28% enhancement in lipid productivity on 4^th^ day. Highest values of lipid productivity of 33% were recorded on 8^th^ day in 0.1% glucose supplemented medium containing sodium thiosulphate. Fatty Acid Methyl Ester (FAME) profiles generated revealed significant reduction in the content of Poly unsaturated fatty acids (PUFA) and enhanced Mono unsaturated fatty acids (MUFA) (especially oleic acid) in the treatments involving tryptophan, Vitamin B12, sodium pyruvate and glucose. This study reveals the promise of using sodium thiosulphate along with selected substrate for enriching the quality and quantity of lipids, which can be valuable for exploiting algae as a source of biodiesel.

**Electronic supplementary material:**

The online version of this article (doi:10.1186/2193-1801-1-33) contains supplementary material, which is available to authorized users.

## Background

The last few decades have seen a growing interest in using microalgae, cyanobacteria and other photosynthetic bacteria as potential producers of renewable fuels, such as biodiesel, biohydrogen and biogas. Biodiesel production from microalgae is a relatively novel concept and these organisms offer the greatest photosynthetic efficiency, as a consequence of a minimum of internally competitive plant functions and limited nutrient requirements, besides exhibiting fast reproductive cycles. The yield of biodiesel from microalgae depends up on both the biomass concentration of the cultures and the oil content of individual cells ( Becker 2004 ; Chisti 2008 ). The total content of lipids in microalgae may vary from about 1–85% of the dry weight (i.e. lipid productivity), with values higher than 40% being typically achieved under stress conditions ( Chisti 2007 ).

Factors such as temperature, irradiance and, most markedly, nutrient availability have been shown to affect both lipid composition and lipid content in several algae ( Takagi and Karseno 2006 ; Rao et al. 2007 ). To develop cost-effective algal oil production, researchers have experimented with photoheterotrophy / mixotrophy and heterotrophy for enhancing lipid productivity, especially with species of *Chlorella* ( Ceron Garcia et al. 2005 ; Schenk et al. 2009 )*.* Recent studies have shown that the global flux distribution in oleaginous *Chlorella protothecoides* and *Chlamydomonas reinhardtii* remains stable under nitrogen limiting conditions and is controlled by the availability of carbon precursors ( Xiong et al. 2010 ; Fan et al. 2012 ). Many microalgae can accumulate lipids due to excess photosynthesis and some species can accumulate high amount of lipids under heterotrophic or environmental stress, such as nutrient deficiency or salt stress ( Jang et al. 2011 ).

The genus *Chlorella* has been a model organism in this context, especially in studies on modulating lipid accumulation, as several strains exhibit heterotrophy ( Miao and Wu 2006 ; Xu et al. 2004 ; Liang et al. 2009 ; Ordog et al. 2012 ). Kay (1991 ) recorded that *Chlorella sorokiniana w*as a promising freshwater non-motile unicellular alga, accumulating high amounts of lipids and proteins. ( Wan et al. 2011 ) analyzed the growth, lipid content and expression levels of three important genes involved in lipid biosynthesis pathway of *Chlorella sorokiniana,* as influenced by mixotrophy and found the organism most suited to mixotrophy, exhibiting 51% lipid content.

Our earlier studies revealed that certain microalgae, especially those belonging to the genus *Chlorella* exhibit enhanced growth and lipid accumulation under light and dark, in the presence of glucose. Among the set of *Chlorella* strains evaluated, *Chlorella sorokiniana* MIC-G5 highest lipid productivity in the presence of 2% glucose, both under mixotrophic and heterotrophic conditions ( Ratha et al. 2012 ). This strain was therefore selected for further in depth analyses in the present investigation.

Researchers have recorded a high lipid content of 55% during heterotrophic growth of *Chlorella protothecoides* and developed efficient processes, combining bioengineering and transesterification for obtaining high quality diesel ( Miao and Wu 2006 ). The point of concern is to identify stimuli which can enhance oil/lipid accumulation in micro algae without affecting their growth rate. The simultaneous operation of photosynthesis and respiration, in the presence of glucose and light is known to lead to more reactive oxygen species, than microalgae can themselves scavenge. The role of reducing agents such as sodium thiosulphate can be useful in this context, as observed in *Chlorella* sp. ( Feng et al. 2005 ). ( Mandal and Mallick 2009 ) reported enhanced lipid accumulation in a *Scenedesmus* strain, in the presence of sodium thiosulphate and glucose. However, other reducing agents have not been evaluated for their role in microalgal lipid accumulation and limited information on this aspect is available in published literature. The citrate synthase representing the pace-making enzyme in the first step of the Citric Acid Cycle (catalyzes the condensation of acetate from acetyl CoA with oxaloacetate to form citrate) is inhibited by high ratios of ATP:ADP, acetyl-CoA:CoA, and NADH:NAD, high concentrations of ATP, acetyl-CoA, and NADH. This is because such metabolic states reveal that the energy supply is high for the cell, hence, our experiments were focused towards addition of metabolic intermediates in the presence of a reducing agent for diverting acetyl CoA to malonyl CoA and thereby towards lipid biosynthesis (Additional file 1: Figure S1).

The objective of the present study was therefore directed towards identifying promising substrate-reducing agent combination which can lead to enhanced lipid quality and productivity in this promising strain of *Chlorella sorokiniana* under mixotrophic conditions.

## Results

Preliminary studies with this organism had shown that that *Chlorella sorokiniana* grown with glucose mixotrophically was most suitable for enhancing lipid productivity ( Ratha et al. 2012 ). The present investigation was undertaken to evaluate further the role of different reducing agents and metabolic intermediates/substrates on lipid content and FAME profiles under mixotrophic conditions.

### Effect on growth and lipid productivity

**Figure 1:**
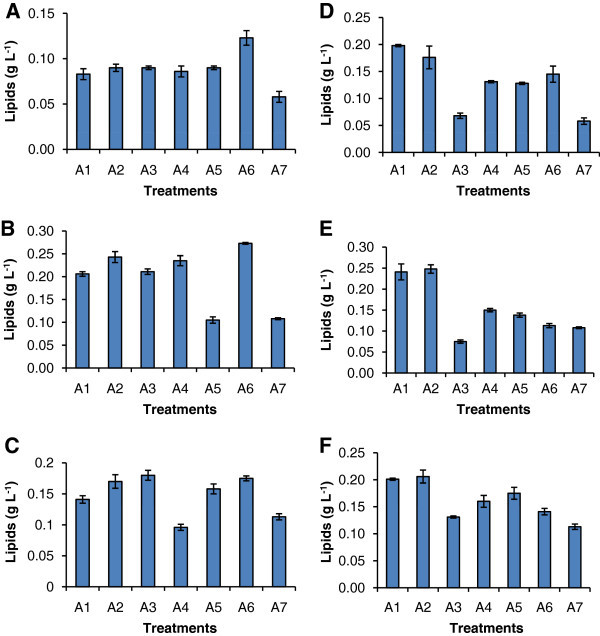
**Lipid accumulation in*Chlorella sorokiniana*MIC-G5 in the presence of reducing agent (Sodium thiosulphate and methyl viologen).** (**A**-**C**) sodium thiosulphate on 4, 8, 12 d and (**D**-**E**) methyl viologen on 4, 8, 12d respectively. A denotes type of treatments; A1 (BBM+sucrose), A2 (BBM+fructose), A3 (BBM+sodium pyruvate), A4 (BBM+tryptophan), A5 (BBM+alanine), A6 (BBM+glucose), A7 (BBM, Control). Mean n = 3 replicates.

The effects of two different reducing agents (sodium thiosulphate and methyl viologen) along with six substrates (three carbon sources- sucrose, fructose and glucose; two amino acids- tryptophan and alanine) and sodium pyruvate on growth is presented in Additional file 2: Table S1. The growth was significantly enhanced upto 8^th^ day in all treatments, with the highest values of 2.16 and 1.63 (Abs_750_) recorded in BBM supplemented with methyl viologen + fructose and sodium thiosulphate + tryptophan on the 8^th^ day of cultivation respectively. The lipid accumulation was evaluated on 4^th^, 8^th^ and 12^th^ day (Figure 1) and the highest lipid content of 0.27 g L^-1^ was observed in the samples grown in sodium thiosulphate supplemented with glucose on 8^th^ day of cultivation. In the methyl viologen treatments, the highest values of 0.248 g/L were recorded with fructose. Sodium thiosulphate increased the lipid productivity from 16% in its control to 39%, when glucose was added.

### Evaluation of sodium thiosulphate and different substrates

**Figure 2:**
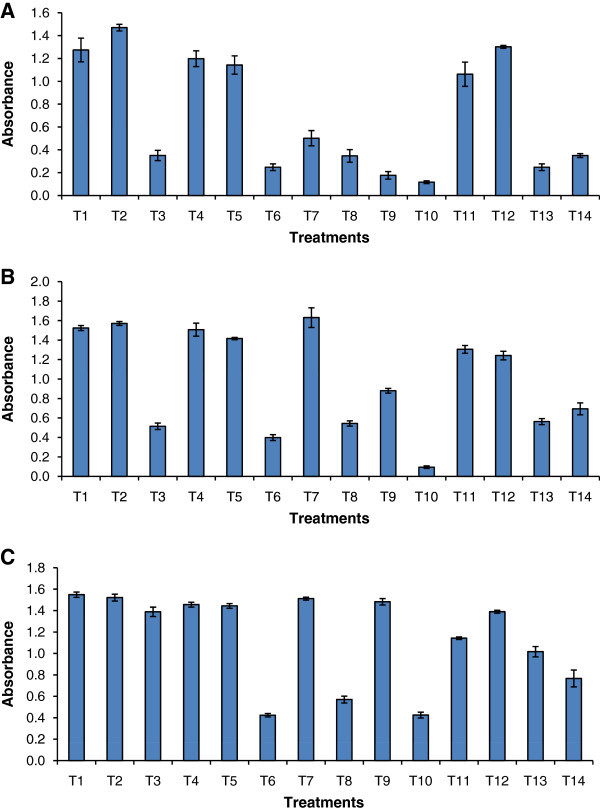
**Growth of*Chlorella sorokiniana*MIC-G5 measured in terms of absorbance (750nm) on 4**^**th**^**, 8**^**th**^**, and 12**^**th**^**d of cultivation in BBM containing sodium thiosulphate (1%) and with different substrates (T); T denote type of substrates; T1 (sucrose), T2 (fructose), T3 (glycine), T4 (glycerol), T5 (sodium pyruvate), T6 (biotin), T7 (tryptophan), T8 (vitamin B12), T9 (leucine), T10 (niacin), T11 (alanine), T12 (glucose), T13 (BBM), T14 (sodium thiosulphate).** Mean n = 3 replicates.

**Figure 3:**
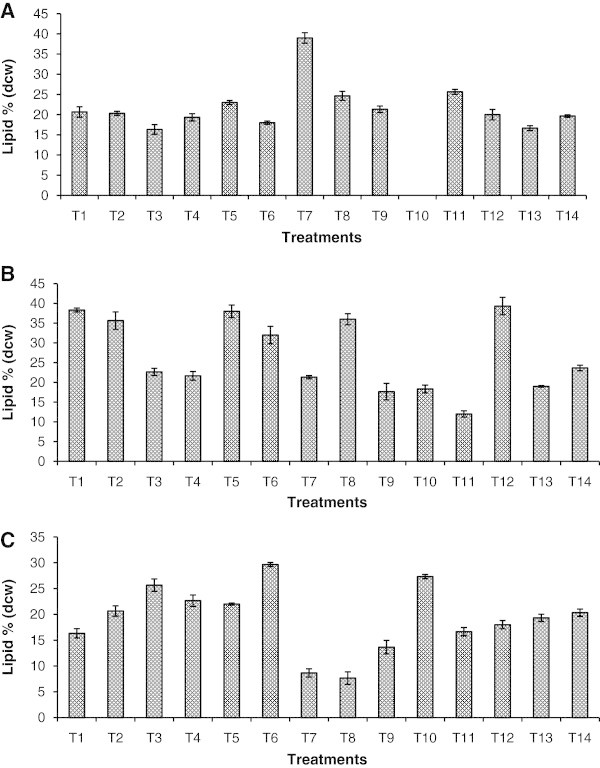
**Lipid accumulation in different treatments (A) on 4**^**th**^**, (B) 8**^**th**^**and (C) 12**^**th**^**day of cultivation in BBM containing sodium thiosulphate (1%) and different substrates - T1 (sucrose ) / T2 (fructose) / T3 (glycine) / T4 (glycerol) / T5 (sodium pyruvate)/ T6 (biotin) / T7 (tryptophan) / T8 (vitamin B12) / T9 (leucine) / T10 (niacin) / T11 (alanine) / T12 (glucose) / T13 (BBM) / T14 (sodium thiosulphate) respectively.** Mean n = 3 replicates.

The effect of sodium thiosulphate with twelve substrates absorbance (Abs 750), chlorophyll and carotenoids contents are presented in Figure 2 and Additional file 2: Table S2. The highest chlorophyll content of 38.76 μg mL^-1^ and 4.8 μg mL^-1^ total carotenoids were recorded on 8^th^ day in sodium thiosulphate supplemented with tryptophan. The lipid productivity (mg g^-1^) in BBM and BBM+ST gradually increased from 160–190 and 19-20% by the 12^th^ day of incubation (Figure 3). The lipid productivity was enhanced in the presence of tryptophan on the 4^th^ day (390 mg g^-1^). Highest values were observed in sodium thiosulphate + glucose supplemented culture, upto 8^th^ day. In niacin supplemented culture, no growth was recorded on the 4^th^ day, but by the 8^th^ and 12^th^ day, 18.33 and 27.33% lipids were recorded. The highest values were recorded in 8^th^ day cultures, in terms of lipid productivity (% DCW) in glucose supplemented treatment (39% or 390 mg g^-1^), and followed by sodium pyruvate (38%) and Vitamin B12 (36%).

### Upscaling of selected promising substrates and effect on lipid productivity and quality

The promising substrates - comprising tryptophan, glucose, sodium pyruvate and Vitamin B12 were taken up further in 5 L flasks containing 2 L medium supplemented with sodium thiosulphate and the selected substrate.

**Figure 4:**
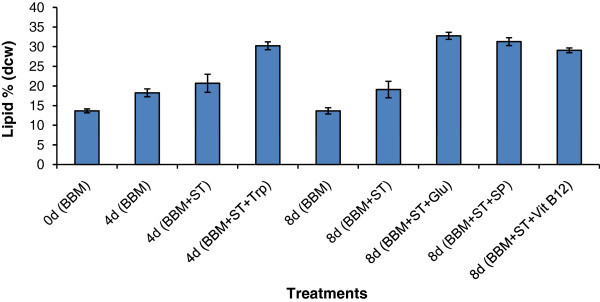
**Lipid productivity of*Chlorella sorokiniana*MIC-G5 in Haffkine flasks.** BBM (Bold’s basal medium), ST (sodium thiosulphate), Trp (tryptophan), Glu (glucose), SP (sodium pyruvate), Vit. B12 (Vitamin B12). Mean n = 3 replicates.

On 4^th^ day, the highest values in terms of turbidity (Abs_750_ 1.26), chlorophyll (8.96 μg mL^-1^) and carotenoids (1.53 μg mL^-1^) were recorded in sodium thiosulphate + tryptophan treatment (Additional file 2: Table S3). The highest values of turbidity (Abs_750_ 1.85), chlorophyll (16.6 μg mL^-1^) were obtained in medium supplemented with sodium thiosulphate alone, but highest lipid content of 0.238 g L^-1^ was recorded in medium supplemented with glucose and sodium thiosulphate on 8^th^ day of cultivation. Cultures supplemented with sodium thiosulphate + vitamin B12 recorded highest total carotenoids (1.46 μg mL^-1^) d on 8^th^ day of cultivation (Additional file 2: Table S4). In terms of lipid productivity (% DCW), medium supplemented with glucose and sodium thiosulphate recorded highest values of 33% and sodium pyruvate (31.33%) on the 8^th^ day, followed by tryptophan (30.33%) on the 4^th^ day (Figure 4).

### Microscopic analyses

**Figure 5:**
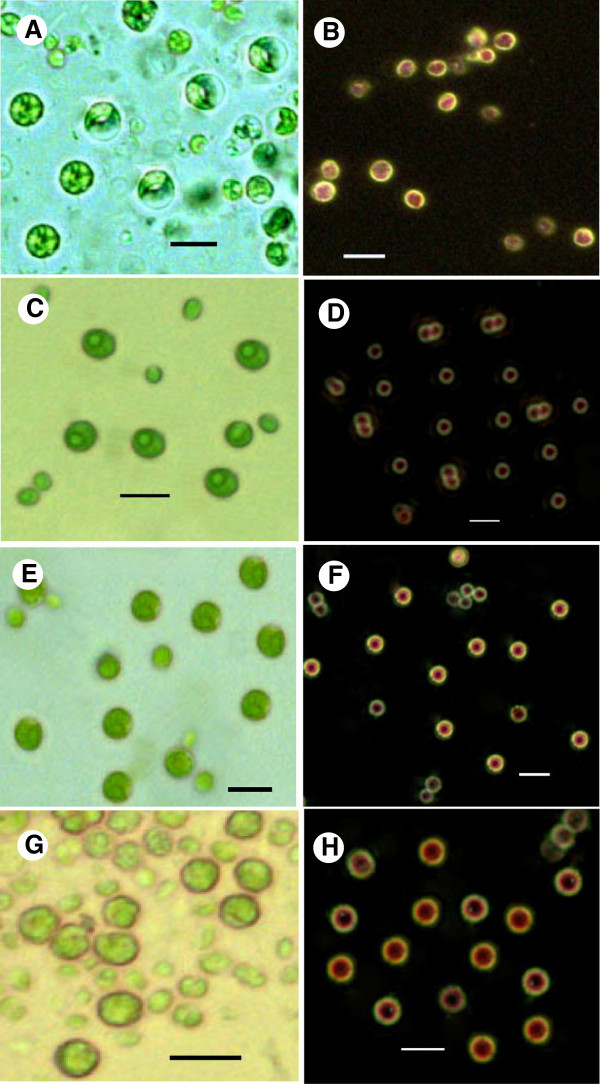
**Light microscopic images (A, C, E and G) and Nile red stained photographs (B, D, F and H) of*Chlorella sorokiniana*MIC-G5, grown in BBM alone (A, B), or supplemented with sodium thiosulphate and Vitamin B**_**12**_**(C, D), or sodium thiosulphate and tryptophan (E, F) or sodium thiosulphate and sodium pyruvate (G, H).**

Light microscopic analyses showed that the cells appeared more robust when grown in BBM with sodium thiosulphate and tryptophan for 4^th^ day and in Vitamin B12/ sodium pyruvate supplemented medium for 8^th^ day (Figure 5 A,C,E,G). Nile red staining clearly illustrated the cell size enlargement and increased lipid content under the influence of tryptophan (Figure 5F) and thiosulphate (Figure 5H). The color of staining was enhanced, as observed in Figure 5 (F) and (H).

### FAME profiles and their analyses

The quantitative analysis of the lipids generated in the form of FAME profiles, revealed that 97.1- 97.7% of the fatty acids belonged to C16-C18 type with USF/SFA ratio in the range of 1.1-2.1 in 4^th^ day and 98.4-98.9% of C16-C18 and ratio between USF: SFA in the range 1.9-2.7 on 8^th^ day of cultivation (Table 1). Highest amount of saturated fatty acids (47.9%) was recorded after growth in BBM + sodium thiosulphate on 4^th^ day. The relative PUFA content ranged from 9.9 to 19 and 8.7 to 24.8 on 4^th^ and 8^th^ day (Tables 1 and 2) respectively. Palmitic acid (16:0) ranged from 25–43.6%, while the linoleic acid (18:2) ranged from 20.2-28.91. In general, the PUFA content reduced in treatments involving only addition of sodium thiosulphate from 60.2% to 42.4% on 4^th^ day and 63.9% to 44.9% on 8^th^ day respectively. The MUFA content increased from 8.0-9.7% and 6.5 to 23.4 % on 4^th^ and 8^th^ day respectively. Addition of tryptophan brought about a 50 and 75% enhancement over sodium thiosulphate supplemented flasks, and control (BBM) on the 4^th^ day. An almost two folds increase in 16:1 and 18:1 fatty acids in sodium thiosulphate + tryptophan treatments, besides 60% enhancement in total lipids (Additional file 3: Figure S2). On 8^th^ day, MUFA content, more than 50% increase was recorded in all the treatments.

**Table 1:** **Qualitative analysis of FAME profiles, in terms of fatty acids (percent on dry cell weight) of*Chlorella sorokiniana*MIC-G5 grown in different treatments on 4**^**th**^**day of cultivation**

Fatty acid	Treatment		
	BBM (C)	BBM+ST	BBM+ST+Trp
12:0	0.3	0.4	0.5
14:0	0.6	1.0	0.6
16:0	29.1	43.6	33.0
16:1	2.7	2.9	7.9
16:2	10.6	9.7	9.8
16:3	6.6	4.0	5.2
18:0	1.5	2.4	1.5
18:1	5.1	6.5	9.8
18:2	25.0	20.2	20.2
18:3	17.1	8.1	9.7
20:0	0.1	0.3	0.2
20:1	0.1	0.0	0.0
20:2	0.6	0.2	1.0
22:0	0.1	0.0	0.1
22:1	0.1	0.3	0.0
22:2	0.3	0.2	0.5
24:0	0.1	0.2	0.0
C16-C18	97.7	97.4	97.1
SFA^a^	31.8	47.9	35.9
MUFA^b^	8.0	9.7	17.7
PUFA^c^	60.2	42.4	46.4
TL^d^	18.0	20.6	30.3
USF:SFA^e^	2.1	1.1	1.8
USF	68.2	52.1	64.1

BBM (Bold’s basal medium), ST (sodium thiosulphate), Trp (tryptophan), ^a^SFA-saturated fatty acids; ^b^MUFA- monounsaturated fatty acids; ^c^PUFA- polyunsaturated fatty acids; ^d^TL- total lipids; ^e^USF:SFA- ratio between unsaturated and saturated fatty acids; USF = (MUFA+PUFA);

The rankings, based on Duncan’s Multiple Range Test, are denoted by superscripts in the relevant tables and graphs, with ‘a’ denoting the highest rank.

**Table 2:** **Qualitative analysis of FAME profiles, in terms of fatty acids (% dry cell weight basis) of*Chlorella sorokiniana*MIC-G5 grown in different treatments on 8**^**th**^**day of cultivation**

Fatty acid	Treatment				
	BBM (C)	BBM+ST	BBM+ST+SP	BBM+ST+Vit.B_12_	BBM+ST+GL
12:0	0.2	0.1	0.2	0.1	0.1
14:0	0.5	0.8	0.5	0.4	0.4
16:0	27.7	30.8	29.3	25.0	27.5
16:1	2.2	2.4	2.1	2.4	2.1
16:2	12.4	10.4	8.1	12.3	6.5
16:3	8.6	3.3	4.1	5.6	3.5
18:0	1.0	2.3	2.9	1.4	3.2
18:1	4.3	16.2	17.5	11.7	21.2
18:2	25.8	26.7	26.2	28.9	28.0
18:3	16.5	6.3	8.4	11.6	6.9
20:0	0.1	0.2	0.2	0.1	0.2
20:1	0.0	0.1	0.1	0.1	0.1
20:2	0.4	0.0	0.1	0.1	0.0
22:0	0.0	0.1	0.1	0.1	0.1
22:1	0.0	0.0	0.0	0.1	0.0
22:2	0.2	0.1	0.0	0.0	0.0
24:0	0.1	0.2	0.2	0.1	0.2
C16-C18	98.5	98.4	98.6	98.9	98.9
SFA^a^	29.6	34.5	33.4	27.2	31.7
MUFA^b^	6.5	18.7	19.7	14.3	23.4
PUFA^c^	63.9	46.8	46.9	58.5	44.9
TL^d^	19.0	20.3	31.3	29.0	33.0
USF:SFA^e^	2.4	1.9	2.0	2.7	2.2
USF	70.4	65.5	66.6	72.8	68.3

BBM (Bold’s basal medium), ST (sodium thiosulphate), SP (sodium pyruvate), GL(D-glucose), Vit.B12 (Vitamin B12); ^a^SFA-saturated fatty acids; ^b^MUFA- monounsaturated fatty acids; ^c^PUFA- polyunsaturated fatty acids; ^d^TL- total lipids; ^e^USF:SFA- ratio between unsaturated and saturated fatty acids; USF = (MUFA+PUFA).

The rankings, based on Duncan’s Multiple Range Test, are denoted by superscripts in the relevant tables and graphs, with ‘a’ denoting the highest rank.

## Discussion

The cultivation of microalgae, which represent excellent sources of fatty acids, proteins and metabolites with diverse types of activity, are among the biotechnological processes that are receiving increasing attention from industries and researchers ( Behrens and Kyle 1996 ; Courchesne et al. 2009 ). Microalgae exhibit a great variability in lipid content; oil content can reach up to 80%, and levels of 20-50% are quite common ( Powell and Hill 2009 ). The fatty acids that are produced by microalgae can be extracted and converted into biodiesel ( Brown and Zeiler 1993 ). However, variations are recorded due to different growing conditions and the methods of extraction of lipid and fatty acids, which has questioned the economic viability and feasibility of microalgae as sources of biodiesel. On the other hand, the ability of microalgae to adapt their metabolism to varying culture conditions provides opportunities to modify, control and thereby maximise the formation of targeted compounds with non-recombinant microalgae. Mixotrophy is one such potential method for high-density microalgae cultivation, as cultures display more efficient utilization of energy for biomass productivity ( Lee et al. 1987 ; Liang et al. 2009 ).

In recent years, in-depth understanding of the many biosynthetic pathways that can be used for the production of biofuel feed stocks or high value bioproducts has emerged, and novel pathways for the production of specific bioenergy carriers are continuously being discovered in a variety of organisms ( Liu et al. 2011b ; Radakovits et al. 2010 ). It is considered feasible to generate highly efficient production of microalgal biomass, without the need for light in inexpensive, well-defined mineral medium, typically supplemented with glucose ( Bumbak et al. 2011 ). Researchers have recorded cell densities of more than 100 g L^−1^ cell dry weight with *Chlorella, Crypthecodinium and Galdieria* species, while controlling the addition of organic sources of carbon and energy in fedbatch mode. *C. sorokiniana* is a non-motile, unicellular freshwater green microalga, which is known to accumulate large amounts of protein and lipid ( Kay 1991 )*. C. sorokiniana* CCTCC M209220 exhibits a rapid growth rate and high oil content when cultured in mixotrophic condition, hence, considered as a promising candidate species for genetic manipulation and enhanced oil yield.

The critical role of Acetyl Co-A, in regulating not only the Kreb’s Cycle, but also as a precursor for fatty acid synthesis is known ( Kim 1983 ; Brennan and Owende 2010 ). Therefore, inclusion of additives/carbon sources which can enhance acetyl CoA/malonyl CoA pool – which represents the central carbon donor for fatty acid synthesis, can be a possible strategy for enhancing lipid productivity. Analyses of global flux distribution in oleaginous *Chlorella protothecoides* revealed that in the presence of glucose, the glyoxalate shunt remains inactive; thereby leading to partitioning of carbon only through TCA ( Xiong et al. 2010 ). Therefore, addition of certain metabolic intermediates/carbon sources etc., in a reducing environment (using sodium thiosulphate/methyl viologen) can help to divert metabolic intermediates to malonyl CoA, which represents the first step of fatty acid synthesis, instead of being used in Kreb’s Cycle. Reducing agents such as sodium thiosulphate are known to protect cells by scavenging reactive oxygen produced as a result of biodegradation of exogenous organic carbon and enhance the lipid pool ( Feng et al. 2005 ). It is well known that an increase in reducing power in the cell can lead to an enhancement in the pool of NADH, and citrate synthase is not functional under such conditions ( Feng et al. 2005 ; Mandal and Mallick, 2009 ). This can lead to diversion of Acetyl CoA to malonyl CoA, thereby increasing lipid pool. Methyl viologen, commonly known as paraquat is a widely employed broad spectrum herbicide, and its toxicity to animals and man is mediated by lipid peroxidation; however its role in lipid accumulation has not been investigated ( Bus Aust and Gibsont 1976 ).

The present study was therefore directed towards understanding the effect of different substrates/metabolic intermediates and reducing agents sodium thiosulphate and methyl viologen) on enhancing lipid productivity of this promising *Chlorella* sp. In the present investigation, comparative growth kinetics and lipid productivity in the presence of two reducing agents- sodium thiosulphate and methyl viologen provided interesting results. Growth studies revealed that tryptophan was most productive in the presence of sodium thiosulphate, but with methyl viologen, fructose performed better. Lipid productivity was significantly higher in tryptophan supplemented cultures with both reducing agents. Sodium thiosulphate is known to play a dual role as a potent antioxidant and chelator of calcium and other toxic substances and is classified by the FDA as a direct food substance affirmed as generally recognized as safe. On the other hand, methyl viologen, undergoes redox cycling *in vivo*, being reduced by an electron donor such as NADPH, before being oxidized by an electron receptor such as dioxygen to produce superoxide, a major ROS (reactive oxygen species). It inhibits photosynthesis, besides being a groove-binding DNA ligand. In the present study, the low concentration used did not inhibit growth or lipid accumulation, but stringent monitoring may need to be employed for using methyl viologen, as compared to the safety of sodium thiosulphate utilization. Also, comparing the lipid accumulation in the presence of both reducing agents, sodium thiosulphate proved more effective, recording the highest values of 0.273 g/L on 8^th^ day.

Although microalgae grow with various carbonaceous compounds, glucose is considered the preferred carbon source, because of its ease of handling, availability and safety ( Lee 2004 ; Perez-Garcia et al. 2011 ; Sun et al. 2008 ). Tryptophan, glycine and yeast extract have also been evaluated for their potential to enhance growth or product formation ( Shen et al. 2010 ). Acetate and ethanol are considered possible alternatives but, because of their respective corrosive effects or high flammability, are only used when an exceptional productivity enhancement is achieved ( de Swaaf et al. 2003 ). Although the cost of (pure) glucose is high for microalgal production, valorisation of the biomass as animal and fish feed supplements, after use of biomass as biodiesel may be promising from an industrial point of view ( Chisti 2007,2008 ; Brennan and Owende 2010 ).

Further experiments were undertaken to evaluate the lipid productivity (expressed as % lipids, on DCW basis) - a product of biomass productivity and lipid content, using 12 different carbon sources/ metabolic intermediates, along with sodium thiosulphate. Supplementation with sodium thiosulphate (1%) only, enhanced the lipid productivity from 16.66 (BBM) to 19.66% on 4^th^ day and 23.66% in 8^th^ day cultures, which is equivalent to 18% and 42% increase over control respectively. On 4^th^ day, highest lipid productivity of 39% was recorded with tryptophan supplementation. Highest lipid productivity of 39.33% in glucose, followed by 38.8% in sucrose, and 38.00% in sodium pyruvate, 35.66% in fructose and 36.0% in vitamin B12 was recorded on 8^th^ day. But, the most significant enhancement in lipid productivity was observed in glucose, which was up to 39.33% on 8^th^ day and in case of tryptophan up to 39.0% on the 4^th^ day. The promising feature recorded was that the increase over control was highest in tryptophan in 4^th^ day (57.26%) as compared to 8^th^ day (10.93%). Dry cell weight or DCW (1086.6μg/ml) and lipid content (255.0 μg/ml) were recorded on 8^th^ day, which represented a significant increase over 8^th^ day control. In the case of glucose, maximum lipid productivity was observed in 8^th^ day which represents an increase of 51.69% over control and 49.14% over 4^th^ day values. In our earlier study, this strain exhibited 32.33% lipid productivity when grown mixotrophically with 2% glucose for 18 days; however, in the present study, an enhancement in lipid productivity to 39.33% was recorded by growing only for 8 days in medium supplemented with 0.1% glucose and 1% sodium thiosulphate.

Among the various carbon substrates, glucose, in particular, is used for the production of high-value compounds, where the processes need to be reproducible for prospective regulatory approval for pharmaceutical manufacture. The ability of a number of microalgal species to grow with organic carbon substrate has been demonstrated previously ( Droop 1974 ). However, the number of current commercially important microalgae that are capable of growth on organic carbon substrates in the dark, and where experience of fedbatch cultivation has been gained, is very limited. The role of carbon substrates lead to different biomass/ substrate yields and also affect the formation of the targeted product through flux distribution is well investigated ( Fan et al. 2012 ). The ability of microalgae to use organic carbon as an energy source is important because it can minimize the inhibitory effects of seasonal and diurnal light limitation on growth in outdoor cultures. A considerable number of algae, for example *Chlamydomonas*, *Spirulina*, *Chlorella*, *Galdieria*, *Scenedesmus*, and *Micractinium*, can grow mixotrophically and heterotrophically in the presence of organic matter such as carbohydrates and acetate ( Brennan and Owende 2010 ; Lee 2004 ). The biochemical composition of microalgae under mixotrophy may not only improve biomass production, but also augment lipid content, which is very important for the production of biodiesel from microalgae ( Xu et al. 2004 ), which was further illustrated in our study.

Majority of research work undertaken on mixotrophic cultivation of microalgae using limited amounts of glucose have shown it to be an effective method to obtain high microalgal biomass productivity and beneficial for increasing lipid and protein accumulation, especially for strains of *Chlorella sorokiniana* ( Wan et al. 2011 ). Enhanced photosynthesis, on addition of glucose has been reported in several algae, including cyanobacteria ( Wang et al. 2000 ). In the present study, all the substrates enhanced chlorophyll upto 8^th^ day of growth, which is in agreement with observations on glucose-grown *Chlorella vulgaris* UAM101 cells, acetate-grown *Chlamydomonas reinhardtii* cells and fructose-grown *Anabaena variabilis* ATCC 29413 cells ( Valiente et al. 1992 ; Courchesne et al. 2009 ). This may be because glucose promoted the donation of electrons to the plastoquinone pool from the respiratory substance, and the transforming of energy was promoted by photosynthetic system, which provided the energy needed by anabolism of cells caused by addition of glucose to the medium ( Wang et al. 2000 ). But few reports revealed that glucose could reduce photochemical efficiency of photosystem II (PS II) and levels of the PS II reaction centre proteins ( Liu et al. 2009 ).

The cell size and lipid content of *Chlorella* cells grown with different substrates were compared using light microscopy and Nile Red staining. Nile red, (9-diethylamino-5H-benzo[α]-phenoxazine-5-one) is a fluorescent hydrophobic dye, that fluoresces intensely, and in a range of colors, when in contact with organic solvents and hydrophobic lipids. Over the past two decades, there have been a number of papers reporting Nile red methods for the determination of intracellular lipids in different organisms. Most of these methods used relative fluorescence intensities to compare or estimate the intercellular lipid contents in these organisms, and they gave correlations between the fluorescence emission and cell number or lipid content ( Chen et al. 2011 ). But in our investigation, light microscopic images with Nile red stain exhibited a good correlation of stain intensity with lipid content. It was found that the cell solidity and size changed after addition of substrates, tryptophan or sodium pyruvate in BBM with sodium thiosulphate. By using low-density cultures of *Botryococcus braunii* under both light and dark culture conditions, ( Tanoi et al. 2011 ) found that both cell and colony size increased significantly, accompanied by increase in the size of oil granules in cells, when the cells were cultured with glucose. This finding was consistent with other *Botryococcus* strains, as recorded by ( Zhang and Kojima^1988^ ) who recorded increase in colony size of *B. braunii* with high light intensity. Glucose seems to play a role in to bringing about changes in oil distribution inside the cells or by enhancing oil accumulation in cells and enlargement of the granule. However, the mechanism underlying the induction of large cell and granule size is unclear and the relationship between cell and granule size and oil content requires further investigation. But, based on our findings and available information, Nile red staining can be proposed as a simple, rapid, and inexpensive sentinel screen for lipids and identifying the potential biodiesel sources.

( Feng et al. 2005 ) were among the earliest researchers to illustrate the utility of appropriate concentrations of sodium thiosulphate and glucose in enhancing lipid accumulation in *Chlorella* sp. They observed an increase in polar lipids in the presence of sodium thiosulphate, which was however modified to similar values as in control on addition of both glucose and sodium thiosulphate. However, in depth analyses of individual classes of fatty acids was not undertaken. Phylogenetic relatives of *Chlorella zofingiensis*, such as *Chlorella vulgaris* and *C. protothecoides* were reported to accumulate high amounts of lipids when cultivated under heterotrophic conditions ( Liu et al. 2011a ; Hsieh and Wu 2009 ). However, knowledge on *Chlorella* species to accumulate lipids and fatty acids under different growth modes remains largely unknown.

( Petkov and Garcia 2007 ) found that the fatty acid composition of 14:0, 16:0; 16:1; 16:2, 16:3, 18:0, 18:1, 18:2 and 18:3 can be a useful marker for identifying *Chlorella.* In our investigation, Fatty acid Methyl ester (FAME) profiles were generated from upscaling experiment with selected promising samples. The analyses revealed highest qualities of saturated fatty acids (47.9%) in medium supplemented only with sodium thiosulphate on 4^th^ day, with a concurrent 15-20% reduction in Poly unsaturated fatty acids (PUFA) and 9% enhanced Mono unsaturated fatty acids (MUFA) content. 16:1 and 18:1 were enhanced from 2.7 to 7.9% and 5.1 to 9.8% respectively, in BBM + sodium thiosulphate + tryptophan. However, relative PUFA values were two folds higher in this treatment as compared to control or BBM + sodium thiosulphate. FAME profiles showed interesting changes in the presence of sodium thiosulphate with/without substrates on the 8^th^ day. PUFA content decreased from 63.9 to 44.9 in BBM+ST+Glucose, while MUFA content enhanced from 6.5 to 19.7 in BBM+ST+sodium pyruvate. Highest total lipids of 33% were recorded in BBM+ST+Glucose, which recorded four folds enhancement in MUFA, along with 33% decrease in PUFA. Our analyses are in consonance with earlier studies ( D’ Oca et al. 2011 ; Liu et al. 2011a^2011^b ) who also reported the predominance of 18:3 and 18:2 fatty acids in *Chlorella* species; additionally in our samples, 16:0 was found to be a major component. The absence of fatty acids with odd number of carbon atoms (related with bacteria) provides proof for an axenic culture being used for experiments and analyses ( Petkov and Garcia, 2007 ).

Unlike photoautotrophic cells, heterotrophic cells are known to channel excessive carbon for the biosynthesis of storage lipids, e.g., neutral lipids, instead of converting carbon into membrane lipids for building photosynthetic apparatus, exhibiting a much higher amount of oleic acid ( Liu et al. 2011a^2011^b ). This in turn, helps to balance the oxidative stability and low-temperature properties and promotes the quality of biodiesel ( Knothe 2009 ). In our investigation, a significant enhancement in oleic acid (18:1) was recorded on both 4^th^ and 8^th^ day, with a fivefold enhancement in BBM+ST+Glucose on 8^th^ day. Wan et al. ( 2011 ) found that *Chlorella sorokiniana* was well suited for lipid production based on its high biomass production rate and lipid content reaching 51% during mixotrophy. Real-time PCR assays revealed increased expression levels of *acc*D (heteromeric acetyl-CoA carboxylase beta subunit), and reduced levels of *rbc* L (ribulose 1, 5- bisphosphate carboxylase/oxygenase large subunit). Similar to higher plants, microalgae synthesize fatty acids in the chloroplast using a single set of enzymes, of which acetyl-CoA carboxylase (ACCase) is a rate-limiting enzyme for fatty acid synthesis while stearoyl ACP desaturase plays an important role in determining the ratio of unsaturated to saturated fatty acids ( Lane et al. 1974 ). Tryptone, glycine and yeast extract have also been evaluated for their potential to enhance growth or product formation ( Shen et al. 2010 ). Amino acids are known to enter the TCA via several intermediates and their inclusion in medium can enhance the Acetyl CoA indirectly. Additionally, in the presence of a reducing agent such as sodium thiosulphate, as recorded in our investigation, a diversion towards malonyl CoA and fatty acid synthesis may have taken place. Furthermore, several microalgae that are grown in pure culture with mineral medium require supplementation with the vitamins ( Droop 2007 ), and the enhancement observed with Vitamin B12 supplementation may be related to enhanced growth.

There is a need for the development of appropriate strategies for exploitation of the flexibility of biomass composition within its upper and lower limits as defined by different culture conditions and/or the altered supply of chemical elements in the culture medium. This as a means of enhancing biomass and/or product formation is one of the major challenges in the area of biofuels. Research efforts worldwide have indicated that this needs to be specific for each algal strain. Our investigation has clearly brought out the promise of using sodium thiosulphate along with selected metabolic intermediates/substrates-glucose, tryptophan, sodium pyruvate and vitamin B12 in modulating significant changes in lipid content and FAME profiles of *Chlorella sorokiniana*, especially, the reduction in PUFA and enhanced oleic acid content which further emphasize their significance for enhanced lipid accumulation and biodiesel production.

## Methods

The axenic culture of green alga *Chlorella sorokiniana* Shih. et Krauss MIC-G5 was obtained from the culture collection of the Division of Microbiology, IARI, New Delhi. The culture was routinely maintained through 2% inoculation into 150 ml Erlenmeyer flasks containing 40 ml Bold’s Basal Medium (BBM). A temperature at 25±2°C under a photoperiod of 16:8 h light and dark at light intensity of 33 μmol photon /m^2/^ s PAR (Photosynthetically Active Radiation) was used for growth. The culture was also grown in BBM supplemented with sodium thiosulphate (1000 ppm / 1% / 63 mM) and methyl viologen (0.01 ppm / 0.00001%), alone and supplemented with six selected substrates- sucrose (2%), fructose (2%), sodium pyruvate (0.1%), tryptophan (0.1%), alanine (0.1%), glucose (0.1%). One selected reducing agent was used further in BBM supplemented with 12 different substrates: sucrose (2%), fructose (2%), sodium pyruvate (0.1%), glycine (0.1%), glycerol (0.1%), biotin (0.1%), tryptophan (0.1%), leucine (0.1%), niacin (0.01%), alanine (0.1%), glucose (0.1%), Vitamin B12 (0.001%). The stock solutions of these compounds were prepared and filter sterilized using 0.22 μm pore size filter membrane, before addition into the autoclaved medium. Preliminary experiments were undertaken to decide the optimal concentration of the substrates used (Momocha 2012). The flasks were hand shaken two to three times daily to maintain proper mixing. Further, the promising combinations were upscaled in 5 L Haffkine flasks, containing 2 L medium and aeration (2 L/min) was provided for effective mixing under stationary conditions. The culture grown in BBM served as control.

### Growth attributes, carotenoids and carbohydrates

The cell concentration was determined by measuring the changes of turbidity in the culture medium (Absorbance at 750 nm: Abs_750_) using a UV–VIS spectrophotometer (Perkin Elmer model Lambda) upto 12^th^ day. Dry cell weight (DCW) was determined gravimetrically using a known amount of algal culture by centrifugation at 3000 g for 10 min. The algal pellet was washed twice with distilled water, and the harvested biomass was dried at 70°C in an oven until it reached a constant weight. To estimate chlorophyll, 10 ml of algal culture was centrifuged at 5000 g for 10 min and the pellet was treated with known volume of methanol and kept in a water bath for 30 min at 60°C. The absorbance of the pooled extracts was measured at 652 and 665 nm for chlorophyll (a + b) and at 470 nm for carotenoids. The concentrations were estimated using standard equations ( Lichtenthaler 1987 ). Chlorophyll, carotenoids and carbohydrates were expressed (%), in terms of dry cell weight (DCW). All the experiments were carried out using triplicate samples.

### Extraction and estimation of lipids

The total lipids were extracted from microalgal biomass using a modified method of Dittmer and Wells (1969 ). The lipids were extracted with mixture of chloroform and methanol (2:1, v/v), and then separated into chloroform and aqueous methanol layers by the addition of methanol and water to give a final solvent ratio of chloroform: methanol: water, 2:2:0.8. The organic layer containing the lipids was washed with 1% NaCl solution, collected and evaporated to dryness under vacuum. Activated charcoal was used to remove all pigments, before lipid content was determined gravimetrically. All the experiments were carried out in triplicate.

### FAME analyses

The fatty acid composition of algal fatty acid methyl esters were determined by modification of the Association of Official Analytical Chemists (AOAC) Official Method 948.15 Fat (Crude) in Seafood, Acid Hydrolysis method, 1995 ( Hungerford 1995 ). Fatty acid methyl esters of the oil were prepared by refluxing the dried sample at 70°C for 3 h in 2% sulphuric acid in methanol. The esters were extracted into ethyl acetate, washed free of acid and passed over anhydrous sodium sulphate. The ethyl acetate extracts were further concentrated using a rotary evaporator. The fatty acid composition was analyzed using an Agilent 6890 N series gas chromatography equipped with FID detector on a split injector. A fused silica capillary column (DB-225, 30 × 0.32 m i.d., J & W Scientifics, USA) was used with the injector and detector temperature maintained at 220°C and 255°C respectively. The oven temperature was programmed at 160°C for 2 min and finally increased to 230°C at 4°C/min. The carrier gas was nitrogen at a flow rate of 1.5 mL/min. The area percentages were recorded with a standard HP Chemstation Data System. Relative PUFA content is expressed as the ratio between the percentages of the different fatty acids: saturated (SATs), monounsaturated (MUFAs) and UFAs, using the formula (PUFA/SAT+MUFA). The unsaturation index was also determined by multiplying the percentage of each fatty acid by the number of double bonds present in the molecule.

### Microscopic analyses

The cells were observed using Zeiss Model Axio Scope. A1, after incubation with different substrates. Nile red (9-(Diethylamino) -5H benzo [α] phenoxazin- 5-one) staining was used to detect intracellular lipid droplets ( Greenspan et al. 1985 ).

### Statistical analyses

The statistical analyses were performed using the software SPSS 10. One-way analysis of variance (ANOVA) was used to evaluate the differences among the treatments. In case, the ANOVA effects were significant, comparisons between the different means were made to quantify and evaluate the source of variation, and CD (Critical Differences) and SEM (Standard error of Means) values were calculated at 0.05 P level. SD (Standard deviation) values are depicted in the graphs as error bars. The superscripts in Tables denote the rankings based on Duncan’s Multiple Range test and different alphabets denote significantly different values, as analyzed using SPSS.

## Electronic supplementary material

Additional file 1: **Figure S1.** Networking of metabolic processes inside the cell. (PPT 170 KB)

Additional file 2: **Table S1.** Comparative growth kinetics of *Chlorella sorokiniana* MIC-G5 in grown in sodium thiosulphate/methyl viologen supplemented with along with substrates. **Table S2.** Chlorophyll and carotenoids of *Chlorella sorokiniana* MIC-G5 grown in BBM containing sodium thiosulphate and different substrates. **Table S3.** Growth, chlorophyll and carotenoids of *Chlorella sorokiniana* MIC-G5 grown in Haffkine flasks with different substrates on 4^th^ day of cultivation. **Table S4.** Growth, chlorophyll and carotenoids of *Chlorella sorokiniana* MIC-G5 grown in under Haffkine flasks with different substrates on 8^th^ day of cultivation. (DOC 61 KB)

Additional file 3: **Figure S2.** Chromatograph depicting FAME profile of Chlorella sorokiniana grown in BBM containing sodium thiosulphate (1%) and tryptophan. (PDF 950 KB)
