# Supplementary material for: Biochemical modulation of growth, lipid quality and productivity in mixotrophic cultures of Chlorella sorokiniana
Source: Springerplus. 2012 Oct 6;1:33. doi: 10.1186/2193-1801-1-33 (PMC3725904; doi:10.1186/2193-1801-1-33)
Supplement: Supplementary file 1 — Additional file 1: Figure S1. Networking of metabolic processes inside the cell. (PPT 170 KB) [file 40064_2012_14_MOESM1_ESM.ppt]

## Slide 1
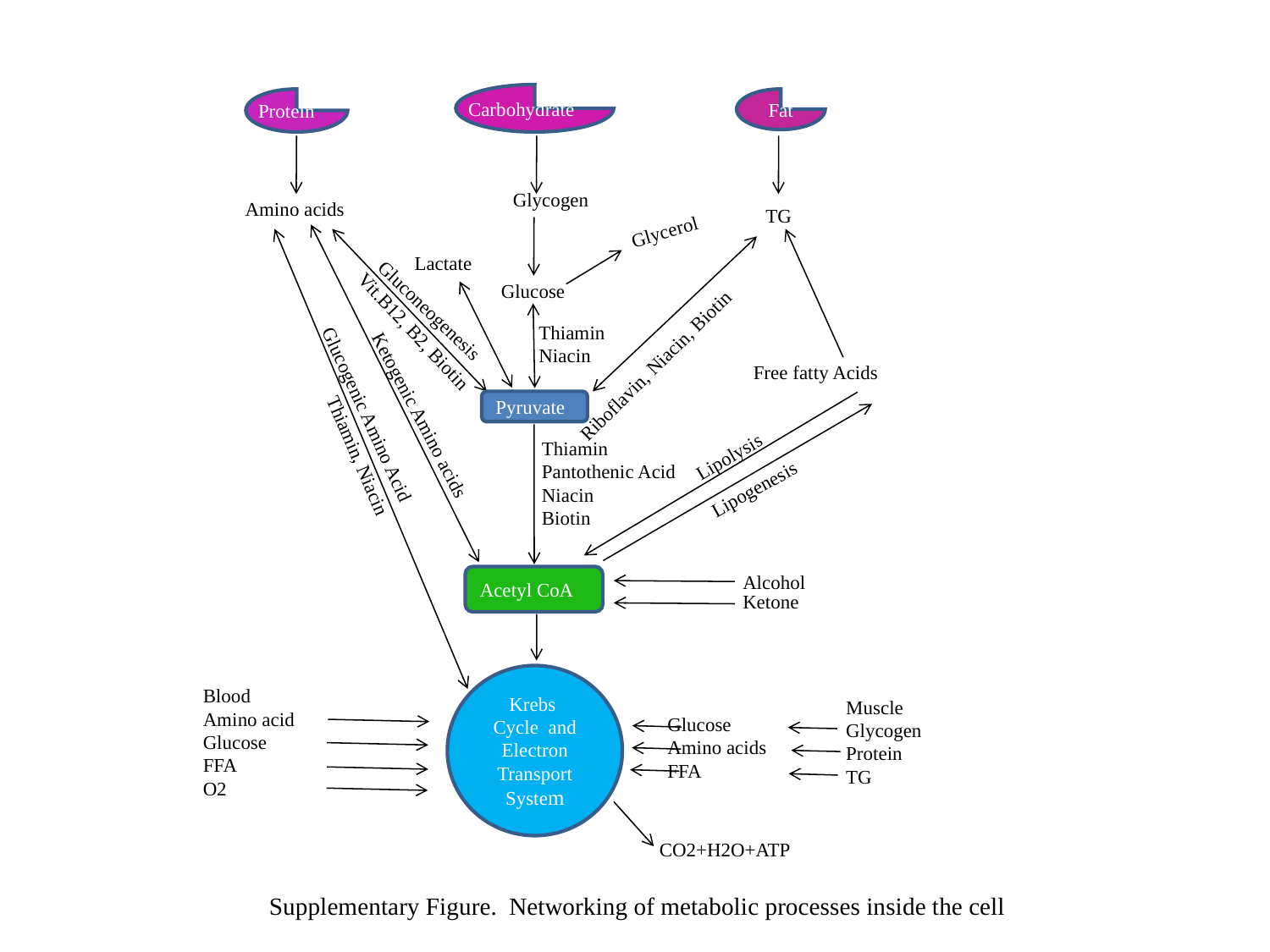

Carbohydrate
Protein
Fat
Glycogen
Amino acids
 TG
Glycerol
Lactate
Glucose
Gluconeogenesis
Thiamin
Niacin
Vit.B12, B2, Biotin
Riboflavin, Niacin, Biotin
Free fatty Acids
Pyruvate
Ketogenic Amino acids
Thiamin
Pantothenic Acid
Niacin
Biotin
Glucogenic Amino Acid
Lipolysis
 Thiamin, Niacin
 Lipogenesis
Alcohol
Acetyl CoA
Ketone
Krebs Cycle and
Electron Transport System
Blood
Amino acid
Glucose
FFA
O2
Muscle
Glycogen
Protein
TG
Glucose
Amino acids
FFA
CO2+H2O+ATP
Supplementary Figure. Networking of metabolic processes inside the cell
