# Supplementary material for: Biochemical modulation of growth, lipid quality and productivity in mixotrophic cultures of Chlorella sorokiniana
Source: Springerplus. 2012 Oct 6;1:33. doi: 10.1186/2193-1801-1-33 (PMC3725904; doi:10.1186/2193-1801-1-33)
Supplement: Supplementary file 2 — Additional file 2: Table S1. Comparative growth kinetics of Chlorella sorokiniana MIC-G5 in grown in sodium thiosulphate/methyl viologen supplemented with along with substrates. Table S2. Chlorophyll and carotenoids of Chlorella sorokiniana MIC-G5 grown in BBM containing sodium thiosulphate and different substrates. Table S3. Growth, chlorophyll and carotenoids of Chlorella sorokiniana MIC-G5 grown in Haffkine flasks with different substrates on 4th day of cultivation. Table S4. Growth, chlorophyll and carotenoids of Chlorella sorokiniana MIC-G5 grown in under Haffkine flasks with different substrates on 8th day of cultivation. (DOC 61 KB) [file 40064_2012_14_MOESM2_ESM.doc]

**Supplementary Table 1**

Comparative growth kinetics of *Chlorella sorokiniana* MIC-G5 in grown in sodium thiosulphate/methyl viologen supplemented with along with substrates.

|  | sodium thiosulphate | | | methyl viologen | | |
| --- | --- | --- | --- | --- | --- | --- |
| Treatments | 4^th^ day | 8^th^ day | 12^th^ day | 4^th^ day | 8^th^ day | 12^th^ day |
| A1 | 1.27^ab^ | 1.54^a^ | 1.55^a^ | 1.52^b^ | 1.88^b^ | 1.65^b^ |
| A2 | 1.47^a^ | 1.57^a^ | 1.55^a^ | 1.74^a^ | 2.16^a^ | 1.94^a^ |
| A3 | 1.14^ab^ | 1.42^b^ | 1.44^b^ | 1.05^e^ | 1.46^d^ | 1.18^d^ |
| A4 | 0.05^c^ | 1.63^a^ | 1.51^a^ | 1.32^c^ | 1.75^bc^ | 1.60^bc^ |
| A5 | 1.06^b^ | 1.31^c^ | 1.09^c^ | 1.20^d^ | 1.23^e^ | 1.47^bc^ |
| A6 | 1.30^ab^ | 1.24^c^ | 1.39^b^ | 1.53^b^ | 1.62^c^ | 1.11^d^ |
| A7 | 0.25^c^ | 0.56^d^ | 1.02^d^ | 0.38^f^ | 0.63^f^ | 1.41^c^ |
| SEM | 0.11 | 0.04 | 0.02 | 0.03 | 0.05 | 0.07 |
| CD (*P* >0.05) | 0.30 | 0.10 | 0.05 | 0.09 | 0.13 | 0.19 |

Superscripts denote rankings based on Duncan’s Multiple Range test and different alphabets denote significantly different values, as analyzed using statistical package.

A denotes type of treatments; A1 (BBM + sucrose), A2 (BBM + fructose), A3 (BBM + sodium pyruvate), A4 (BBM + tryptophan), A5 (BBM + alanine), A6 (BBM + glucose), A7 (BBM). Mean n = 3 replicates.

**Supplementary Table 2**

Chlorophyll and carotenoids of *Chlorella sorokiniana* MIC-G5 grown in BBM containing sodium thiosulphate and different substrates.

| Substrates | Chlorophyll (µg mL^-1^) | | | Carotenoids (µg mL^-1^) | | |
| --- | --- | --- | --- | --- | --- | --- |
|  | 4^th^ day | 8^th^ day | 12^th^ day | 4^th^ day | 8^th^ day | 12^th^ day |
| T1 | 2.87^de^ | 85.66^d^ | 4.00^f^ | 0.90^de^ | 1.23^cdef^ | 0.60^ef^ |
| T2 | 3.03^de^ | 10.23^c^ | 2.93^f^ | 0.37^fg^ | 1.70^cd^ | 0.60^ef^ |
| T3 | 4.03^cd^ | 10.33^c^ | 7.77d^e^ | 0.23^g^ | 1.63^cde^ | 1.60^cd^ |
| T4 | 2.50^e^ | 7.47^de^ | 2.93^f^ | 1.60^b^ | 1.03^efg^ | 0.60^ef^ |
| T5 | 2.43^e^ | 7.43^de^ | 2.53^f^ | 2.13^a^ | 1.70^cd^ | 0.33^f^ |
| T6 | 2.47^e^ | 2.77^g^ | 3.47^f^ | 0.50^fg^ | 0.67^fg^ | 0.73^ef^ |
| T7 | 5.03^c^ | 38.76^a^ | 25.33^a^ | 0.90^de^ | 4.80^a^ | 4.03^a^ |
| T8 | 3.53^de^ | 6.93^e^ | 1.03^d^ | 1.13^cd^ | 1.63^cde^ | 1.83^bcd^ |
| T9 | 3.63^de^ | 7.60^de^ | 17.13^b^ | 0.27^g^ | 1.13^def^ | 2.30^bc^ |
| T10 | 1.47^f^ | 4.07^fg^ | 11.70^c^ | 0.27^g^ | 0.43^g^ | 2.17^bc^ |
| T11 | 11.57^a^ | 17.36^b^ | 18.17^b^ | 1.60^b^ | 2.47^b^ | 2.10^bc^ |
| T12 | 7.60^b^ | 8.00^de^ | 6.13^e^ | 1.33^bc^ | 1.87^c^ | 1.23^de^ |
| T13 | 3.60^de^ | 5.476^f^ | 13.47^c^ | 0.67^ef^ | 1.33^cde^ | 2.43^b^ |
| T14 | 3.23^de^ | 4.63^f^ | 7.77^e^ | 0.30^g^ | 1.00^efg^ | 1.73^bcd^ |
| SEM | 0.45 | 0.46 | 0.68 | 0.11 | 0.20 | 0.24 |
| CD (*P* >0.05) | 1.24 | 1.274 | 1.88 | 0.31 | 0.55 | 0.66 |

Superscripts denote rankings based on Duncan’s Multiple Range test and different alphabets denote significantly different values, as analyzed using statistical package
T denotes treatments, T1 (sucrose), T2 (fructose), T3 (glycine), T4 (glycerol), T5 (sodium pyruvate), T6 (biotin), T7 (tryptophan), T8 (vit. B12), T9 (leucine), T10 (niacin), T11 (alanine), T12 (glucose), T13 (BBM), T14 (sodium thiosulphate).

**Supplementary Table 3**

Growth, chlorophyll and carotenoids of *Chlorella sorokiniana* MIC-G5 grown in Haffkine flasks with different substrates on 4^th^ day of cultivation.

| Treatment | Growth | Chlorophyll  (µg mL^-1^) | Carotenoids  (µg mL^-1^) |
| --- | --- | --- | --- |
| BBM | 0.581^c^ | 5.30^c^ | 1.23^a^ |
| BBM+ST | 1.102^b^ | 7.46^b^ | 1.20^a^ |
| BBM+ST+Trp | 1.262^a^ | 8.96^a^ | 1.53^a^ |
| SEM | 0.005 | 0.169 | 0.006 |
| CD(*P* >0.05) | 0.013 | 0.468 | 0.016 |

Superscripts denote rankings based on Duncan’s Multiple Range test and different alphabets denote significantly different values, as analyzed using statistical package

0 day: Growth (0.074); Chlorophyll (1.10); Carotenoids (0.03).

BBM (Bold’s basal medium), ST (sodium thiosulphate), Trp (tryptophan).

**Supplementary Table 4**

Growth, chlorophyll and carotenoids of *Chlorella sorokiniana* MIC-G5 grown in under Haffkine flasks with different substrates on 8^th^ day of cultivation.

| Treatment | Growth | Chlorophyll  (µg mL^-1^) | Carotenoids  (µg mL^-1^) |
| --- | --- | --- | --- |
| BBM | 1.212^d^ | 12.16^c^ | 0.80^a^ |
| BBM+ST | 1.846^a^ | 16.60^a^ | 0.93^a^ |
| BBM+ST+Glu | 1.792^b^ | 8.23^e^ | 0.83^a^ |
| BBM+ST+SP | 1.523^c^ | 1.05^d^ | 1.30^a^ |
| BBM+ST+Vit B12 | 1.523^c^ | 13.56^b^ | 1.46^a^ |
| SEM | 0.004 | 0.22 | 0.22 |
| CD(*P* >0.05) | 0.013 | 0.61 | 0.60 |

Superscripts denote rankings based on Duncan’s Multiple Range test and different alphabets denote significantly different values, as analyzed using statistical package
0 day: Growth (0.074); Chlorophyll (1.10); Carotenoids (0.03).
BBM (Bold’s basal medium), ST (sodium thiosulphate), Glu (glucose),

SP (sodium pyruvate), Vit B12 (Vitamin B12).
