# Supplementary material for: Biochemical modulation of growth, lipid quality and productivity in mixotrophic cultures of Chlorella sorokiniana
Source: Springerplus. 2012 Oct 6;1:33. doi: 10.1186/2193-1801-1-33 (PMC3725904; doi:10.1186/2193-1801-1-33)
Supplement: Supplementary file 3 — Additional file 3: Figure S2. Chromatograph depicting FAME profile of Chlorella sorokiniana grown in BBM containing sodium thiosulphate (1%) and tryptophan. (PDF 950 KB) [file 40064_2012_14_MOESM3_ESM.pdf]

Data File C:\HPCHEM\1\DATA\SA\IARI-C1.D Sample Name: C FAME

===

Injection Date	: 3/21/2012 9:01:22 PM		Seq. Line :	3
Sample Name	: C FAME chlorella sp. BBM + Sodium Thiosulphate + Trp (4th day)		Location :	Vial 203
Acq. Operator	: MADHU		Inj :	1
Acq. Instrument	: Instrument 1		Inj Volume :	2 µl
Acq. Method	: C:\HPCHEM\1\METHODS\DB225.M			
Last changed	: 3/21/2012 6:21:29 PM by MADHU			
Analysis Method	: C:\HPCHEM\1\METHODS\DB225.M			
Last changed	: 3/24/2012 11:51:39 AM by SARIKA (modified after loading)			

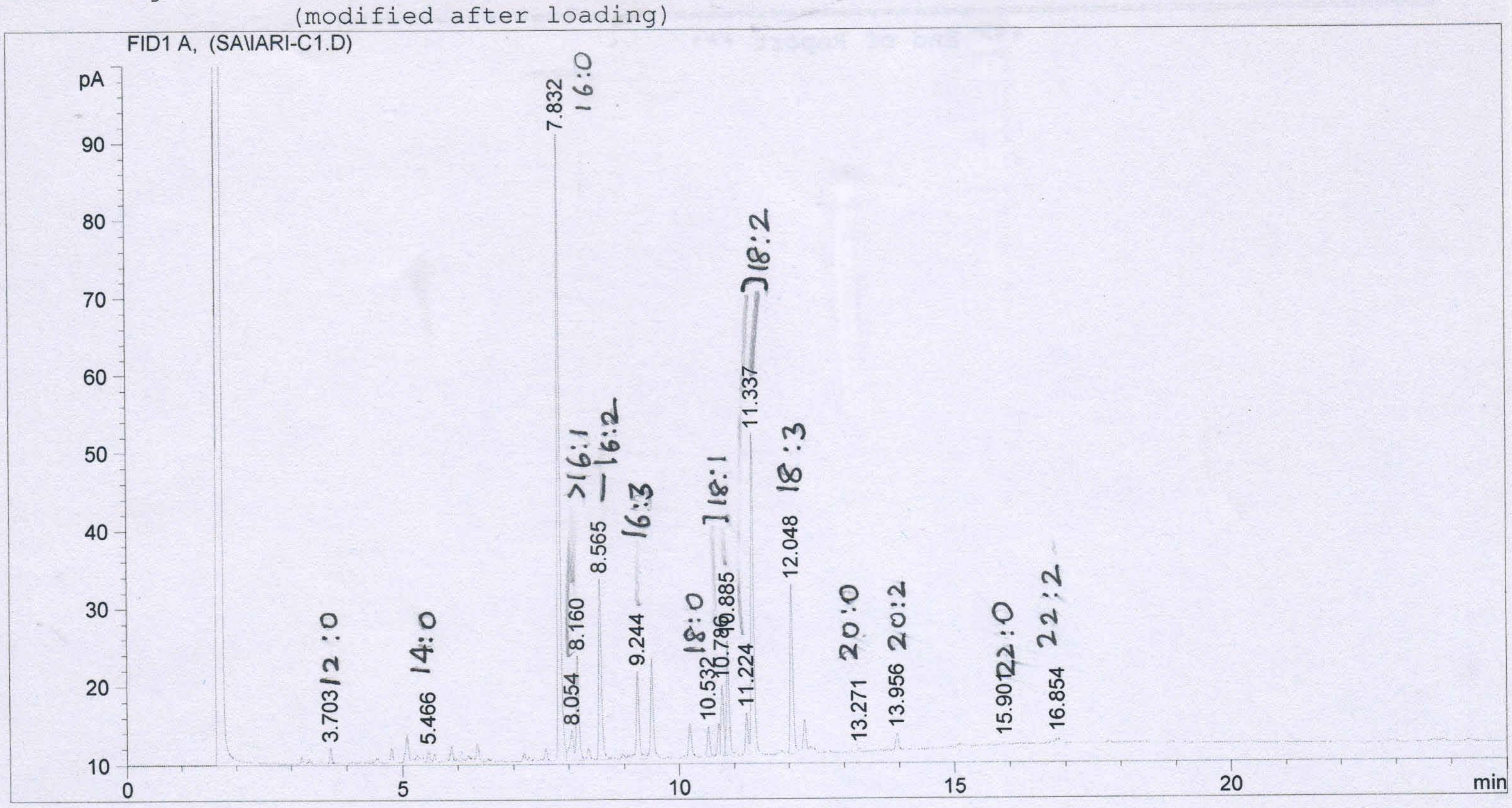

===

Area Percent Report

===

Sorted By	:	Signal
Multiplier	:	1.0000
Dilution	:	1.0000

Use Multiplier & Dilution Factor with ISTDs

Signal 1: FID1 A,

Peak #	RetTime [min]	Type	Width [min]	Area [pA*s]	Height [pA]	Area %	
1	3.703	BB	0.0392	4.72207	1.88892	0.59797	12:0
2	5.466	BV	0.0476	4.63677	1.51998	0.58717	14:0
3	7.832	BV	0.0498	260.28680	80.26655	32.96095	16:0
4	8.054	VV	0.0714	18.27079	3.71463	2.31369	16:1
5	8.160	VV	0.0510	44.28304	13.26271	5.60770	
6	8.565	VB	0.0509	77.16542	23.11693	9.77170	16:2
7	9.244	VB	0.0570	41.00816	11.13119	5.19299	16:3
8	10.532	BB	0.0489	11.90144	3.76648	1.50712	18:0
9	10.786	VV	0.0502	29.22565	8.93167	3.70094	18:1
10	10.885	VB	0.0507	48.05696	14.48589	6.08561	
11	11.224	BV	0.0514	16.83326	5.25741	2.13165	18:2
12	11.337	VB	0.0546	142.76286	41.00827	18.07852	
13	12.048	BB	0.0534	76.67740	21.62199	9.70990	18:3
14	13.271	BP	0.0542	1.53435	4.44777e-1	0.19430	20:0
15	13.956	BB	0.0566	7.52102	2.05772	0.95241	20:2

Peak #	RetTime [min]	Type	Width [min]	Area [pA*s]	Height [pA]	Area %
16	15.901	PP	0.0535	1.00173	2.95821e-1	0.12685
17	16.854	BP	0.0775	3.79473	7.19741e-1	0.48054

Totals : 789.68246 233.49067

Results obtained with enhanced integrator!

*** End of Report ***
